# Optimizing pain management in breast cancer care: Utilizing ‘All of Us’ data and deep learning to identify patients at elevated risk for chronic pain

**DOI:** 10.1111/jnu.13009

**Published:** 2024-07-26

**Authors:** Jung In Park, Steven Johnson, Lisiane Pruinelli

**Affiliations:** ^1^ Sue & Bill Gross School of Nursing, University of California Irvine California USA; ^2^ Institute for Health Informatics, University of Minnesota Minneapolis Minnesota USA; ^3^ College of Nursing and College of Medicine, University of Florida Gainesville Florida USA

**Keywords:** All of Us, breast cancer, cancer pain, chronic pain, deep learning

## Abstract

**Purpose:**

The aim of the study was to develop a prediction model using deep learning approach to identify breast cancer patients at high risk for chronic pain.

**Design:**

This study was a retrospective, observational study.

**Methods:**

We used demographic, diagnosis, and social survey data from the NIH ‘All of Us’ program and used a deep learning approach, specifically a Transformer‐based time‐series classifier, to develop and evaluate our prediction model.

**Results:**

The final dataset included 1131 patients. We evaluated the deep learning prediction model, which achieved an accuracy of 72.8% and an area under the receiver operating characteristic curve of 82.0%, demonstrating high performance.

**Conclusion:**

Our research represents a significant advancement in predicting chronic pain among breast cancer patients, leveraging deep learning model. Our unique approach integrates both time‐series and static data for a more comprehensive understanding of patient outcomes.

**Clinical Relevance:**

Our study could enhance early identification and personalized management of chronic pain in breast cancer patients using a deep learning‐based prediction model, reducing pain burden and improving outcomes.

## INTRODUCTION

Breast cancer represents a significant health challenge for women in the United States, being the most diagnosed cancer with an estimated 298,790 new cases in 2023 alone (American Cancer Society, 2022; Center for Disease Control and Prevention, 2022). A prevalent and debilitating symptom among cancer patients is chronic pain, affecting approximately 35% of individuals (American Cancer Society, 2019). This issue is particularly acute in female breast cancer survivors who are more susceptible to pain (Gallaway et al., 2020). Characterized by pain persisting for more than 3 months, chronic pain severely impacts the quality of life and challenges cancer treatment. The high incidence and profound impact of chronic pain highlights the critical need for effective management strategies and a deeper understanding to enhance the overall outcomes and quality of life for patients diagnosed with breast cancer (Jensen et al., 2010).

Chronic pain in cancer patients is influenced by a confluence of factors, such as clinical conditions, psychosocial elements, and socioeconomic aspects (Van Den Beuken‐Van Everdingen et al., 2016). The clinical conditions directly affect pain intensity and characteristics (Caraceni & Shkodra, 2019). Psychosocial factors, such as stress, anxiety, and depression, can intensify the experience of pain (Riba et al., 2019). Socioeconomic aspects are also known to be significant, as access to healthcare resources, financial stability, and social support systems can greatly impact pain management and overall treatment outcomes (Peppercorn et al., 2011). Therefore, a holistic approach in cancer care is essential, one that encompasses and addresses the complex interplay of clinical, psychosocial, and socioeconomic factors in each patient's experience of pain (Ferrell et al., 2017).

In this context, the ‘All of Us’ program by the NIH emerges as a groundbreaking resource for cancer research (All of Us Research Program Investigators, 2019). This extensive dataset, comprising health data from over a million participants across the United States, incorporates demographic, clinical, environmental, socioeconomic, and lifestyle factors. It offers an in‐depth view of the complex aspects of cancer and its associated chronic pain. The detailed and diverse nature of the ‘All of Us’ dataset is invaluable for developing precise, personalized chronic pain management strategies, improving treatment outcomes and enhancing the quality of life for cancer patients.

In this study, we used data from the ‘All of Us’ program to identify breast cancer patients at high risk for chronic pain, with the goal of optimizing pain management. Specifically, we used an innovative deep learning approach to analyze the large, complex, national‐level ‘All of Us’ dataset. Given recent advantages in artificial intelligence (AI), deep learning techniques hold great promise in cancer research, as they can process vast amounts of data, including time‐series information, to discern patterns and predictors of health outcomes. Tailoring pain management strategies to individual needs through our prediction model could revolutionize our understanding and nursing care of chronic pain in breast cancer patients, resulting in more effective, patient‐focused clinical decisions and an improved quality of life for these individuals.

There have been some studies on predicting chronic pain among breast cancer survivors. However, these either used small samples, relied solely on clinical information, or were conducted outside the United States (Liukas et al., 2023; Lötsch et al., 2018; Tan et al., 2023; Yin et al., 2023). To date, no study has been conducted using a deep learning approach that leverages time‐series data and national‐level health data with socioeconomic information to predict patients at high risk for chronic pain among breast cancer patients. To address this gap, we have leveraged a transformer‐based approach (Vaswani et al., 2017) in deep learning to develop a more accurate and personalized prediction model for chronic pain risk.

## METHODS

### Data sources

This is a retrospective study designed using the ‘All of Us’ Research Program's Controlled Tier Dataset. The dataset is de‐identified and available to authorized users on the Researcher Workbench. Ranging from 03/1980 to 05/2022, the dataset includes demographics, patient survey, lab measurement, drug exposure, and diagnosis information. Although the ‘All of Us’ Program Dataset provides various data types, such as biosamples and physical measurements, only a limited number of populations had all that information. To ensure a sufficient sample size for our deep learning approach, we exclusively included data types available for the majority of breast cancer patients in the dataset. We specifically extracted data for patients with a breast cancer diagnosis (ICD‐9: 170.X; ICD‐10‐CM: C50.X), focusing on their demographic profiles, survey responses, and diagnostic codes. Since the data were fully de‐identified, the study was exempt from Institutional Review Board review at the University of California, Irvine.

### Prediction and outcome variable

Our input variables used for modeling were derived from multiple data elements available in the ‘All of Us’ dataset, primarily including (1) demographic information, (2) diagnosis codes prior to the cancer diagnosis, and (3) survey data. The demographic information encompassed age at the time of breast cancer diagnosis, race, and ethnicity. Moreover, we incorporated diagnosis codes recorded before the breast cancer diagnosis to capture the patients' health status, pre‐existing conditions, overall health, and medical history. We extracted a time‐series of diagnosis codes and the corresponding timestamps (days until the cancer diagnosis) for each patient from the source records. Additionally, patient survey data, which included patient‐reported outcomes and socioeconomic factors, were also included.

Our outcome variable was the occurrence of chronic pain following the cancer diagnosis. To determine the development of new chronic pain and the exacerbation of pre‐existing chronic pain in breast cancer patients, we applied a set of criteria (Table 1) to determine the presence of chronic pain within 3 years following the cancer diagnosis.

**TABLE 1:** Definition of chronic pain in this study.

	Definition
Development of new chronic pain	The presence of chronic pain was identified using ICD‐9‐CM, ICD‐10‐CM, and SNOMED codes recorded after the cancer diagnosis. For ICD‐10‐CM codes, records where the code started with ‘G89’ were selected. For ICD‐9‐CM, records beginning with ‘338.2’ were included. For SNOMED, records with the code ‘82423001’ were selected.
	Alternatively, chronic pain was determined by the use of pain medication* for more than 3 months following the cancer diagnosis, characterized by both of the following conditions: Documentation of consecutive administration of pain medication within a 3‐month period. The total duration of pain medication administration exceeding 3 months.
Exacerbation of Pre‐existing Chronic Pain	This was identified if pain intensity (severity) scores were available before and after the cancer diagnosis, and there was a documented increase in the score.

* Pain medication: Buprenorphine, Butorphanol, Codeine, Dihydrocodeine, Fentanyl, Hydrocodone, Hydromorphone, Levorphanol tartrate, Meperidine hydrochloride, Methadone, Morphine, Opium, Oxycodone, Oxymorphone, Pentazocine, Tapentadol, Tramadol.

### Data preparation and feature selection

The data preparation phase involved tailored processes for each data type—demographic, diagnosis codes, and social survey data—ensuring their compatibility with the deep learning model by mapping the code data with unique integers and tabular data with standard encoding methods. These steps were crucial in converting raw data from the ‘All of Us’ dataset into a format amenable to deep learning analysis, particularly for a Transformer‐based model. By using dedicated encoding techniques for each data type, we ensured that our model could effectively learn from and interpret the complex and diverse information in the dataset.

The demographic data were a standard tabular data, encoded into a numeric form with conventional techniques. For race and ethnicity, we applied one‐hot encoding, creating binary variables for each category to capture this categorical information without introducing ordinal assumptions. Age at diagnosis was normalized by dividing it by 100. This normalization step puts the age variable on a scale that is more consistent with the other variables in the model, improving the model's ability to learn from this feature.

Diagnosis codes were prepared through a two‐step process: concatenating code vocabularies to form unique identifiers and tokenizing these into non‐zero integers for efficient model processing. We managed variable code lengths by setting a standard input sequence length, truncating longer sequences, and padding shorter ones. This approach of truncation and zero‐padding was consistently applied to the timestamp series (Devlin et al., 2018). Despite the categorical nature of these codes, we avoided code mapping (e.g., ICD‐9‐CM to ICD‐10‐CM) or grouping (e.g., 530.81 and 530.8 to 530). This was possible due to a Transformer‐based model's proficiency in handling unmodified, complex data structures—a key advantage of advanced deep learning algorithms.

For the survey data, we used one‐hot encoding (Hancock & Khoshgoftaar, 2020) for each question‐answer pair. This technique converted the categorical survey responses into a binary matrix, facilitating their integration into the predictive model. This approach was particularly important for handling the diverse range of questions and answers within the socio‐economic and health‐related survey data.

Feature selection is often used in deep learning to choose only the most relevant features to reduce the noise in the data, avoid overfitting, and ultimately improve the model performance. For the demographic and survey data, we applied a variance thresholding method prior to the model training to select relevant features from the raw data. Variance thresholding (Guyon & Elisseeff, 2003) is a technique used to filter out features with low variance, as these may not contribute significantly to the predictive power of the model. This thresholding approach ensures that only features with a variance above the specified threshold, accounting for variations in binary attributes, are retained. It streamlines the input data for our model, focusing on attributes that exhibit meaningful variation while efficiently handling binary features.

### Model architecture

We utilized a state‐of‐the‐art Transformer‐based time‐series classifier (Rasmy et al., 2021), specifically tailored to handle the complexities of diagnosis code series within the context of binary classification. This architecture has advantages in capturing the temporal dynamics and patterns in sequential medical data, which is vital for understanding the progression of health events in breast cancer patients.

The Transformer is a type of neural network architecture that has revolutionized the field of natural language processing and has since found applications in various other domains, including health informatics (Vaswani et al., 2017). It is the backbone of the latest Large Language Models (LLMs; Thirunavukarasu et al., 2023) and Generative AI innovations such as BERT (Devlin et al., 2018) and GPT (Brown et al., 2020), and Med‐BERT (Rasmy et al., 2021). The Transformer's core innovation lies in its use of self‐attention mechanisms, which allow the model to weigh the significance of different parts of the input data differently. The self‐attention mechanism computes attention scores, represented in a matrix, to determine how much emphasis to give to each part of the input in the context of the sequence. This mechanism is a key for handling tasks where understanding the relationship between different parts of the data, irrespective of their position in the sequence, is crucial.

**FIGURE 1:**
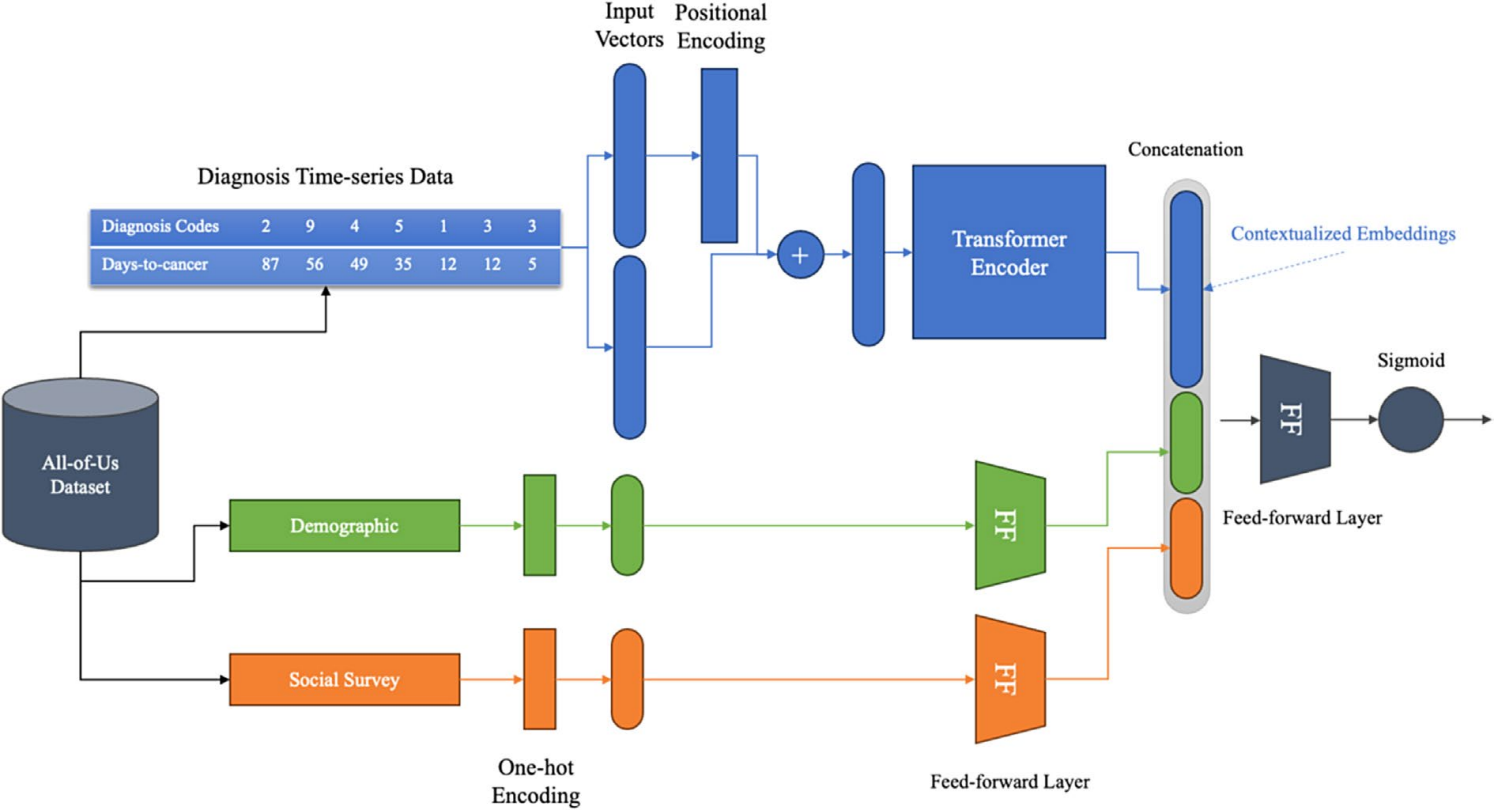
Overview of our transformer‐based chronic pain prediction model: Three different data inputs are passed into dedicated encoders to generate a final vector that can be classified into a single value.

Transformers offer several advantages over traditional sequence processing models, such as RNNs (Recurrent Neural Networks) and LSTMs (Long Short‐Term Memory networks) (Sherstinsky, 2020). One key advantage is their ability to handle long‐range dependencies effectively, meaning they can remember and utilize information from earlier in the sequence. This capability is critical in contexts such as medical histories, where past diagnoses can be highly relevant to current health outcomes. Additionally, Transformers are more parallelizable than RNNs and LSTMs, leading to faster training times and enabling the processing of longer sequences more efficiently (Karita et al., 2019; Zeyer et al., 2019). Figure 1 illustrates the overview of our model architecture.

#### Encoder‐only transformer structure

We used the encoder part of the Transformer architecture for binary classification of time‐series data. Unlike sequence‐to‐sequence models needing encoders and decoders, our model predicts a single variable. The encoder produces contextualized embeddings from diagnosis codes, capturing complex data relationships.

#### Positional encoding and days‐to‐cancer timestamps

Positional encoding and days‐to‐cancer timestamp embeddings were integrated into our Transformer model to enhance the interpretation of diagnosis sequences. The former provides positional context, whereas the latter quantifies the temporal distance to the breast cancer diagnosis. It offers insights into the sequence order, frequency, and timing of patient visits and health events. This dual‐embedding approach significantly enriches the model's analytical capabilities.

#### Incorporating demographic and survey data

Our model architecture merges time‐series diagnosis codes with static demographic and survey data, utilizing feedforward linear layers to process each data type. These layers generate feature vectors, encoding vital information such as demographic data, and socio‐economic factors from survey responses. This integration strategy amplifies the model's predictive capability by combining dynamic and static data sources.

#### Concatenation with contextualized embeddings

After generating feature vectors for demographic and survey data, we concatenate these with the contextualized embeddings from the diagnosis code series. This merges temporal diagnosis information with static demographic and survey data, forming a comprehensive feature set that encapsulates time‐series and static elements, offering a complete perspective of each patient's medical and socio‐economic profile.

#### Final feedforward layer

The model combines diverse feature representations and processes them through a final feedforward layer, producing outputs for binary classification of chronic pain risk. A sigmoid layer then converts these outputs into probabilities, reflecting the likelihood of chronic pain development within 3 years post‐breast cancer diagnosis. By integrating both dynamic and static data, the model harnesses a comprehensive range of predictors, enhancing its accuracy and providing a nuanced understanding of chronic pain factors in breast cancer patients. This demonstrates the Transformer‐based model's capability in managing complex health informatics data.

### Model training and evaluation

We allocated our dataset into three segments: 70% for training, 5% for validation, and 25% for testing. The training set was crucial for the primary training phase, the validation set helped in fine‐tuning the model parameters and mitigating overfitting, and the test set was reserved for evaluating the model's final performance. The model was trained with specific hyperparameters to optimize its performance. To evaluate the performance, we used accuracy, Area Under the Receiver Operating Characteristic Curve (AUROC), precision, and recall metrics.

To determine which input features or variables have the most significant contribution on the model's predictions, we measured the feature importance. We used permutation, measured by the decrease in a model's performance when the values of a single feature are randomly shuffled, thus disrupting the feature‐target relationship. This process, repeated for each feature, identifies the most significant ones based on the largest performance drop – revealing the most influential features.

## RESULTS

### Sample characteristics

The final dataset comprised 1131 patients, with 199 (17.59%) samples representing patients with chronic pain (positive case) and 932 (82.40%) samples representing patients without chronic pain (negative case). We analyzed the data characteristics and compared the differences between positive cases and negative cases. The average age of the group is 57.8 years, with a standard deviation of 10.4 years. The ages range from a minimum of 23.9 to a maximum of 86.8 years. In terms of racial demographics, the majority of the individuals are White, accounting for 997 people (88.2%). Black or African American individuals account for 84 people (7.5%), followed by Asians with 24 people (2.1%). The categories ‘More than one population’ and ‘Other’ include 13 individuals each, representing 1.1% of the total for each group. In terms of ethnicity, Non‐Hispanic or Latino patients were more than 98%.

From the diagnosis records, we identified the top 10 most common diagnosis codes for positive and negative cases, as shown in Table 2. The dataset contained 5740 unique diagnosis codes. From the survey data, we found 81 questionnaires related to patient‐reported outcomes and socioeconomic factors.

**TABLE 2:** Top 10 common diagnosis codes.

All		Positive		Negative	
ICD‐9‐CM 272.4: Other and unspecified hyperlipidemia	280	ICD‐9‐CM 272.4: Other and unspecified hyperlipidemia	60	ICD‐9‐CM 272.4: Other and unspecified hyperlipidemia	219
ICD‐9‐CM 401.9: Unspecified essential hypertension	254	ICD‐9‐CM 401.9: Unspecified essential hypertension	51	ICD‐9‐CM 401.9: Unspecified essential hypertension	202
ICD‐9‐CM 729.5: Pain in limb	196	ICD‐9‐CM 530.81: Esophageal reflux	44	ICD‐9‐CM 729.5: Pain in limb	157
ICD‐10‐CM I10: Essential (primary) hypertension	188	ICD‐9‐CM 733.90: Disorder of bone and cartilage, unspecified	43	ICD‐10‐CM I10: Essential (primary) hypertension	153
ICD‐9‐CM 530.81: Esophageal reflux	179	ICD‐9‐CM 729.5: Pain in limb	38	ICD‐10‐CM E78.5: Hyperlipidemia, unspecified	135
ICD‐9‐CM 733.90: Disorder of bone and cartilage, unspecified	171	ICD‐10‐CM I10: Essential (primary) hypertension	34	ICD‐9‐CM 530.81: Esophageal reflux	134
ICD‐10‐CM E78.5: Hyperlipidemia, unspecified	159	ICD‐9‐CM 272.0: Pure hypercholesterolemia	30	ICD‐9‐CM 244.9: Unspecified acquired hypothyroidism	130
ICD‐9‐CM 244.9: Unspecified acquired hypothyroidism	159	ICD‐9‐CM 465.9: Acute upper respiratory infections of unspecified site	29	ICD‐9‐CM 733.90: Disorder of bone and cartilage, unspecified	127
ICD‐9‐CM 272.0: Pure hypercholesterolemia	137	ICD‐9‐CM 311: Depressive disorder, not elsewhere classified	28	ICD‐9‐CM 780.79: Other malaise and fatigue	110
ICD‐9‐CM 719.46: Pain in joint, lower leg	136	ICD‐9‐CM 719.46: Pain in joint, lower leg	28	ICD‐9‐CM 719.46: Pain in joint, lower leg	107

### Data preparation and feature selection

Despite the high number of unique diagnosis codes, no remapping or grouping was applied; instead, we leveraged a Transformer‐based model's capability to process complex data without simplification. We selected the input sequence length to be slightly higher (128) than the mean length of the codes (109), and then truncated the codes if the sequence was longer. If the number of codes were less than 128, we added padding token (“PAD”, which is defined as 0) to specify that it is padded. The same rule was applied to the time stamp series as well (truncation and zero padding). In our thresholding process for feature selection, we tuned a parameter denoted as ‘p’, which represents the allowable probability of a constant value in binary features. We set ‘p’ to 0.9 for demographic features and 0.8 for survey features due to the lower variance in the demographic feature dataset compared with the survey features.

### Model training and evaluation

The choice of hyperparameters in our model was driven by an empirical testing to balance model complexity with computational efficiency and performance. Specifically, the number of attention heads (4) and encoder layers (2) were chosen to capture sufficient contextual relationships within the data without overcomplicating the model, which can lead to overfitting. The dimension of the feedforward network (32) and the hidden dimensions for demographic and social data (4) were selected to ensure a model that can process features efficiently while maintaining enough capacity to learn significant patterns. We used a constant learning rate of 0.001 throughout the training process, together with Adam optimizer (Kingma & Ba, 2014). The number of epochs was set to 20, providing a balance between adequate learning and preventing overfitting. The positional weight (3.0) was specifically set to mitigate the impact of class imbalance within our dataset. Each parameter was iteratively adjusted to optimize the trade‐off between accuracy and generalization capability using the validation set.

For the model's regularization strategies, we incorporated dropout layers within the transformer encoder, setting a dropout rate of 0.1. Additionally, we adjusted the neural network sizes to further mitigate overfitting. The layer dimensions for the demographic and social components were established at 4. Moreover, to address class imbalance in the dataset, we applied a positive weight of 3.0.

**FIGURE 2:**
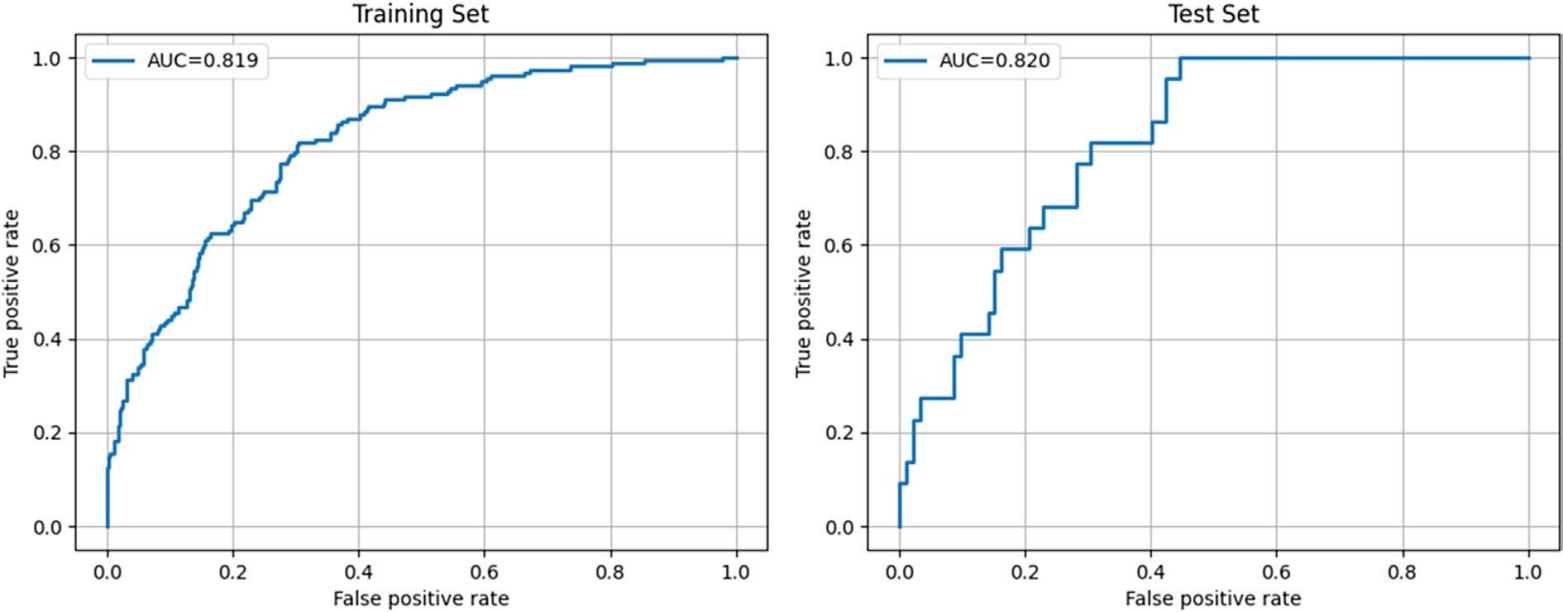
ROC curves from training and test datasets.

**FIGURE 3:**
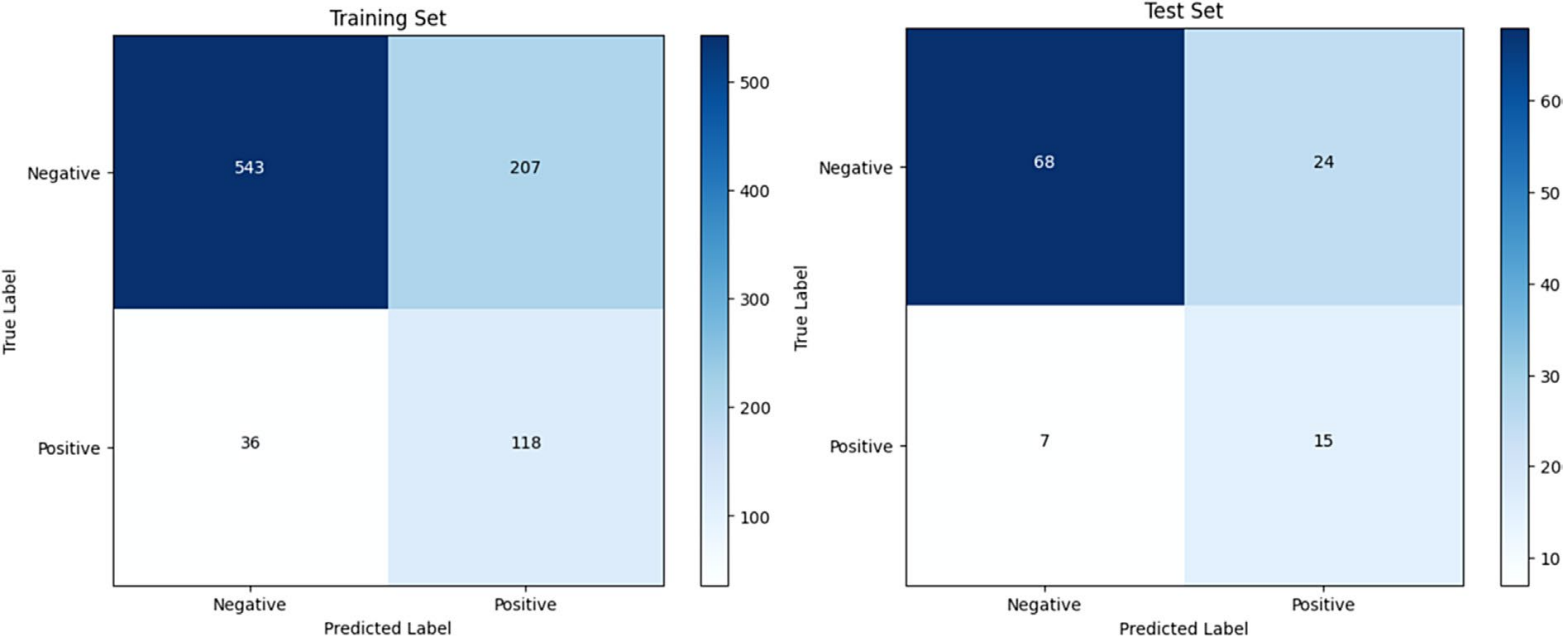
Confusion matrix from both training and test sets.

Regarding the performance evaluation, the accuracy was 73.1% for the training set and 72.8% for the test set. The AUROC was 82.0% for both the training and test sets. Precision was 35.8% for the training set and 38.4% for the test set. In terms of recall, it reached 75.9% for the training set and 68.4% for the test set. Figure 2 presents the ROC curves for the training and test sets. We also computed the confusion matrix to detailed view of the prediction evaluation which is shown in Figure 3.

For further analysis and experiment on the impact of the imbalanced dataset and our mitigation, we explored different positive weight values to refine our model's performance using the training set. Our aim was to achieve a balanced outcome among AUROC, precision, and recall. Initially, with a positive weight of 1.0, the model demonstrated an AUROC of 78.2% and relatively high precision at 72.0%, but the recall was low at 19.5%. When we increased the positive weight to 3.0, the model achieved an improved AUROC of 82.0% and a higher recall of 75.9%, though precision dropped to 35.8%. Finally, with the positive weight further increased to 5.0, the recall reached 96.1% at the cost of a reduced AUROC of 77.4% and precision of 24.2%. Based on these results, we decided to use a positive weight of 3.0 to strike a balance between recall performance and the overall predictive metrics of AUROC and precision. This strategy enabled the model to identify most positive cases effectively while reducing false negatives, aligning with our goal to maintain a reasonable equilibrium between precision and recall.

### Self‐attention mechanism and feature importance

**FIGURE 4:**
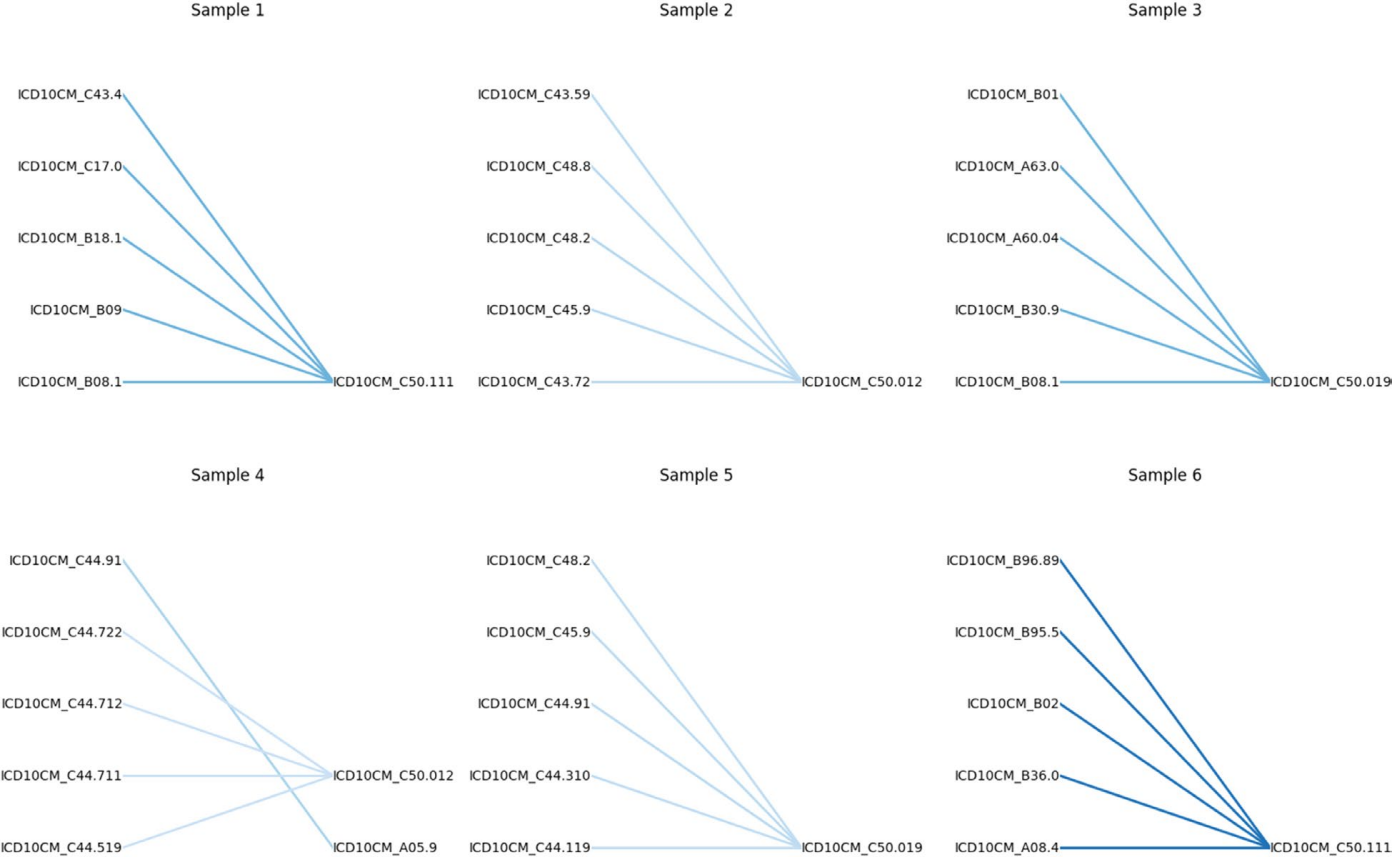
Top 5 strongest connections from the self‐attention mechanism.

Figure 4 illustrates the self‐attention mechanism within a sequence of input codes, highlighting the top five most influential connections. This visualization helps to clarify which parts of the sequence the model views as most important for its predictions, offering insights into the internal reasoning of the neural network and revealing key relationships in the data. Table 3 explains the strongest connections in Figure 4. Consequently, we determined that ‘age at cancer diagnosis’ is the most significant demographic feature, with an importance score of 0.139. Table 4 details the top 10 influential survey question‐answer pairs that affected the prediction performance the most.

**TABLE 3:** Strong connections among diagnosis.

Diagnosis codes	Highly related to
Malignant neoplasm of duodenum Chronic viral hepatitis B without delta‐agent Malignant melanoma of scalp and neck Molluscum contagiosum Unspecified viral infection characterized by skin and mucous membrane lesions	Malignant neoplasm of central portion of right female breast
Malignant neoplasm of peritoneum, unspecified Mesothelioma, unspecified Malignant melanoma of left lower limb, including hip Malignant neoplasm of overlapping sites of retroperitoneum and peritoneum Malignant melanoma of other part of trunk	Malignant neoplasm of nipple and areola, left female breast
Molluscum contagiosum Viral conjunctivitis, unspecified Anogenital (venereal) warts Herpesviral vulvovaginitis	Malignant neoplasm of nipple and areola, unspecified female breast
Basal cell carcinoma of skin, unspecified Squamous cell carcinoma of skin of right lower limb, including hip Basal cell carcinoma of skin of right lower limb, including hip Basal cell carcinoma of skin of unspecified lower limb, including hip Basal cell carcinoma of skin of other part of trunk	Bacterial foodborne intoxication, unspecified Malignant neoplasm of nipple and areola, left female breast
Basal cell carcinoma of skin, unspecified Mesothelioma, unspecified Malignant neoplasm of peritoneum, unspecified Basal cell carcinoma of skin of unspecified parts of face	Malignant neoplasm of nipple and areola, unspecified female breast
Unspecified streptococcus as the cause of diseases classified elsewhere Other specified bacterial agents as the cause of diseases classified elsewhere Viral intestinal infection, unspecified Pityriasis versicolor	Malignant neoplasm of central portion of right female breast

**TABLE 4:** Top 10 influential survey question‐answer pairs.

Question	Answer	Feature importance
How often does a doctor or nurse act as if he or she thinks you are not smart when you go to a doctor's office or other health care provider?	Rarely	0.606
How often are you treated with less respect than other people when you go to a doctor's office or other health care provider?	Rarely	0.296
How often do you have someone who understands your problems?	Most of the time	0.289
Many shops, stores, markets or other places to buy things I need are within easy walking distance of my home. Would you say that you…	Strongly disagree	0.287
In your day‐to‐day life, how often are you treated with less courtesy than other people?	Less than once a year	0.272
How much you agree or disagree that people in your neighborhood generally get along with each other?	Agree	0.271
How much you agree or disagree that people in your neighborhood can be trusted?	Strongly agree	0.267
How often do you feel that you are spiritually touched by the beauty of creation?	Many times a day	0.254
In your day‐to‐day life, how often do you receive poorer service than other people at restaurants or stores?	Never	0.254
How much you agree or disagree that you are always having trouble with your neighbors?	Disagree	0.249

## DISCUSSION

Although chronic pain is prevalent among breast cancer survivors and significantly impacts their quality of life, predicting patients at elevated risk for chronic pain is challenging due to the multifaceted nature of the influencing factors. There is a clear need for a predictive tool that leverages innovative techniques and fully capitalizes on large, diverse datasets. In this study, we utilized advanced deep learning and the versatility of Transformer‐based algorithms to address this need.

### Main contributions

One of the key innovations in our study was the seamless integration of time‐series diagnosis history with static demographic and survey data, creating a comprehensive prediction model. By combining these diverse data sources into a unified model, we aimed to capture a holistic view of each patient's medical and socioeconomic context. This approach allowed us to consider the content of diagnosis codes and the temporal patterns and static attributes that influence chronic pain outcomes.

Additionally, the application of the Transformer architecture in our study is an innovative step, leveraging its powerful attention mechanisms and handling of sequential data to derive meaningful insights from complex medical histories. Our method is distinct in integrating time‐series data that captures the chronological sequence of diagnoses; this approach not only incorporates demographic data, as explored in TransformEHR (Yang et al., 2023), but also introduces survey data. This unique combination fosters a detailed analysis of the various factors contributing to the onset or worsening of chronic pain among breast cancer patients. Through an analysis of the self‐attention matrices within our Transformer‐based model, we gained valuable insights into the diagnosis codes that had the most significant impact on our predictions. This interpretability aspect of our model provided a window into the complex relationships and dependencies within the medical history of breast cancer patients. Identifying these influential codes is a crucial step toward enhancing our understanding of the factors contributing to chronic pain outcomes.

Furthermore, to further dissect the contributions of demographic and social survey data, we used permutation importance which computes the contribution of a feature by random shuffling (Altmann et al., 2010). This analysis allowed us to pinpoint the most impactful features within these datasets. It highlighted the socio‐economic factors and demographic attributes that play a pivotal role in predicting chronic pain in breast cancer patients.

### Modeling results

From the diagnosis records, we identified distinct characteristics in the top 10 most common diagnosis codes for positive and negative cases. Certain diagnoses, such as ICD‐9‐CM 311 for Depressive Disorder, not elsewhere classified, were unique to the top 10 in positive cases. This observation supports existing research, suggesting that depression can amplify pain perception, and increase sensitivity to pain. Conversely, chronic pain can induce depressive symptoms due to ongoing discomfort, diminished quality of life, and the constraints it places on daily activities (Linton & Bergbom, 2011; Surah et al., 2014).

In terms of evaluation, our predictive model demonstrated commendable performance, achieving an AUROC of 82.3%. The model exhibited reasonable recall (68.4%), ensuring that it effectively identified patients at risk of chronic pain. However, we observed a lower precision rate (38.4%), which can be attributed to the relatively small and imbalanced dataset (1:4.7). This imbalance poses a challenge in distinguishing true positive cases from false positives. The difference of evaluation results between training and test sets are small, showing that the regularizations were effective.

Interpreting the strongest connections from the self‐attention mechanism of a neural network, as visualized in Figure 4, involves understanding how different parts of the input sequence relate to each other and contribute to the model's output. The top connections can provide a narrative or justification for the model's decisions. It appears that when a malignant neoplasm, other than breast cancer, is paired with malignant neoplasm of the breast, there is a high correlation with the outcome. This suggests that comorbid cancer or a history of other cancer significantly impacts the risk of chronic pain (Jensen et al., 2010; Paice et al., 2016). Furthermore, when infection is combined with malignant neoplasm of the breast, it exerts a significant impact on the outcome. This suggests a potential link between infections and the development or exacerbation of chronic pain conditions.

### Limitations and future directions

This study has several limitations. First, the demographics of the dataset lacked diversity. The majority of the patients were White (88.2%) and non‐Hispanic (98%), which may limit the generalizability of the findings. This indicates the need for careful consideration in addressing these disparities. Potential approaches could include curating the dataset with an adequate number of underrepresented populations, testing with different populations, or developing race/ethnicity‐specific models when sufficient data are available. Such efforts are crucial to ensure the applicability and relevance of the study's conclusions across diverse groups. Second, although we only included demographics, diagnosis codes, and survey data related socioeconomic status due to limited resources, there are more factors that influence pain than the factors included in this study. Future study is required that includes additional factors such as medications and genetics data. Finally, we only included patients whose survey data was available, which may have introduced a selection bias into our model's predictions. This limitation could potentially impact the representativeness and diversity of our dataset, thus affecting the model's ability to generalize its predictions across a broader patient population. Although we did not explicitly encounter issues with low questionnaire response rates, we recognize the importance of addressing this potential source of bias. Future investigations should consider strategies such as data imputation techniques (Efron, 1994) to account for incomplete data, thereby ensuring the robustness and accuracy of the model's predictions.

## CONCLUSION

Our research represents a significant step forward in predicting chronic pain among breast cancer patients for pain management and effective nursing care, highlighting the power of deep learning and Transformer‐based models. The unique aspect of our model is its holistic integration of both time‐series and static data, enhancing the comprehensive understanding of patient outcomes. However, future work is needed to incorporate additional factors that influence chronic pain, alongside more diverse demographic data. By expanding the application of this deep learning approach across different medical contexts, we aim to uncover new insights and address unresolved questions in healthcare.

### CLINICAL RESOURCES

‘All of Us’ Program Research Hub: https://www.researchallofus.org/data‐tools/workbench/. NIH Office of Data Science Strategy: https://datascience.nih.gov.

## Data Availability

The data that support the findings of this study are available in the NIH's ‘All of Us’ Research Program at https://workbench.researchallofus.org/, Controlled Tier Dataset. These data were derived from the following resources available in the public domain: https://www.researchallofus.org.
